# The Effects of Disease Models of Nuclear Actin Polymerization on the Nucleus

**DOI:** 10.3389/fphys.2016.00454

**Published:** 2016-10-07

**Authors:** Leonid A. Serebryannyy, Michaela Yuen, Megan Parilla, Sandra T. Cooper, Primal de Lanerolle

**Affiliations:** ^1^Department of Physiology and Biophysics, University of Illinois at ChicagoChicago, IL, USA; ^2^Institute for Neuroscience and Muscle Research, Kids Research Institute, The Children's Hospital at WestmeadSydney, NSW, Australia; ^3^Faculty of Medicine, Discipline of Pediatrics and Child Health, University of SydneySydney, NSW, Australia

**Keywords:** nuclear actin, nuclear filaments, skeletal actin mutations, intranuclear rod myopathy, cofilin rods, RNA polymerase II, chromatin, nuclear structure

## Abstract

Actin plays a crucial role in regulating multiple processes within the nucleus, including transcription and chromatin organization. However, the polymerization state of nuclear actin remains controversial, and there is no evidence for persistent actin filaments in a normal interphase nucleus. Further, several disease pathologies are characterized by polymerization of nuclear actin into stable filaments or rods. These include filaments that stain with phalloidin, resulting from point mutations in skeletal α-actin, detected in the human skeletal disease intranuclear rod myopathy, and cofilin/actin rods that form in response to cellular stressors like heatshock. To further elucidate the effects of these pathological actin structures, we examined the nucleus in both cell culture models as well as isolated human tissues. We find these actin structures alter the distribution of both RNA polymerase II and chromatin. Our data suggest that nuclear actin filaments result in disruption of nuclear organization, which may contribute to the disease pathology.

## Introduction

Actin is necessary for maintaining cell structure and driving cell movement and contraction (Dominguez and Holmes, 2011). The capacity of actin to be both a structural protein and generate force comes from actin's ability to dynamically polymerize and depolymerize into polymers and filaments of different lengths. Actin also translocates into the nucleus (de Lanerolle and Serebryannyy, 2011; Dopie et al., 2012). Yet, unlike cytoskeletal actin, actin in the nucleus of somatic cells does not polymerize into persistent filaments. Instead, studies suggest nuclear actin exists as monomers and highly dynamic polymers (McDonald et al., 2006). Nevertheless, nuclear actin has been implicated in a variety of processes including regulation of transcription factors (Rajakylä and Vartiainen, 2014), the nuclear lamina (Simon and Wilson, 2011), DNA damage repair (Andrin et al., 2012; Belin et al., 2015), transcription by RNA polymerase (RNAP) I, II, and III (Hofmann et al., 2004; Hu et al., 2004; Philimonenko et al., 2004), hnRNP binding (Percipalle et al., 2009), and is a necessary component of multiple chromatin remodeling complexes (Bremer et al., 1981; Kapoor and Shen, 2014; Serebryannyy et al., 2016a). Intriguingly, instances of long-range nuclear movement of chromatin and nuclear compartments have been reported to be dependent on nuclear actin polymerization (Forest et al., 2005; Chuang et al., 2006; Wang et al., 2006; Dundr et al., 2007; Hu et al., 2008; Mehta et al., 2010; Chang et al., 2011; Khanna et al., 2014). Collectively, nuclear actin seems to be important in regulating gene accessibility, transcription, and post-transcriptional regulation (de Lanerolle and Serebryannyy, 2011).

Despite multiple studies suggesting nuclear actin is able to polymerize (Amankwah and De Boni, 1994; reviewed in de Lanerolle and Serebryannyy, 2011; de Lanerolle, 2012), there are few documented instances of physiological actin filaments in the nucleus. While in *Xenopus* oocytes nuclear actin filaments form a structural network (Clark and Rosenbaum, 1979; Feric and Brangwynne, 2013; Samwer et al., 2013), there is little direct evidence for a comparable actin-based structure in the normal mammalian nucleus. Fluorescence recovery after photobleaching experiments and actin probes using cloned actin binding domains have indirectly visualized a population of short nuclear actin polymers that exist throughout the nucleus. Additionally, recent reports have indicated that transient nuclear actin filaments are able to form in response to serum stimulation and cell spreading via a formin-mediated mechanism (Baarlink et al., 2013; Plessner et al., 2015). These reports suggest physiological nuclear actin polymerization occurs transiently and their formation of nuclear actin filaments is tightly regulated. However, the role of nuclear actin polymers and transient filaments are still largely a mystery.

Two distinct nuclear actin structures are found in a number of disease states: phalloidin-stainable nuclear actin filaments and cofilin/actin rods. Phalloidin reportedly binds a site accessible in right-handed actin filaments (Dancker et al., 1975; Barden et al., 1987; Drubin et al., 1993; Visegrády et al., 2005). The affinity and specificity of phalloidin binding to actin has made it a widely used marker for actin filaments. Cofilin is commonly considered an actin depolymerizing factor; however, cofilin binding to actin changes its conformation (McGough et al., 1997; Ghosh et al., 2004; Andrianantoandro and Pollard, 2006) and has been shown to form unconventional actin filaments (Nishida et al., 1987). These left-handed cofilin/actin rods are generally not recognized by phalloidin, thus the labeling of actin filaments with phalloidin or incorporation of cofilin indicate different actin structures (Yonezawa et al., 1988; McGough et al., 1997; Kudryashov et al., 2006).

Cofilin/actin rods, which are usually reversible (Nishida et al., 1987; Iida et al., 1992), have been well documented in both the cytoplasm and the nucleus (Bamburg and Wiggan, 2002; Hofmann, 2009). Formation of nuclear cofilin/actin rods has been noted during a variety of cellular stresses including heatshock (Welch and Suhan, 1985; Iida et al., 1986; Nishida et al., 1987), changes in salt concentrations (Iida and Yahara, 1986), overactive cAMP production (Osborn and Weber, 1984), DMSO treatment (Fukui and Katsumaru, 1980; Sanger et al., 1980; Nishida et al., 1987), ATP depletion (Pendleton et al., 2003), actin depolymerization with actin drugs (Yahara et al., 1982), trifluoperazine treatment (Osborn and Weber, 1980), electrical stimulation (Seïte et al., 1973), and adenosquamous cell carcinoma of the axillary sweat glands (Fukuda et al., 1987). Intriguingly, nuclear cofilin/actin rod formation has been implicated in Huntington's disease, whereby abnormal huntingtin protein expression can stabilize the formation of these structures (Munsie et al., 2011). There is also evidence for nuclear microfilament formation in aging neurons, among other diseases (Fiori, 1987; Bamburg et al., 2010). Nevertheless, it remains unclear why these rods form and what their function may be. Individually, nuclear actin and cofilin have both been documented to regulate transcription (Hofmann et al., 2004; Obrdlik and Percipalle, 2011), yet the effects of nuclear cofilin/actin rods have not been studied in the context of transcription or chromatin organization.

Like cofilin/actin rods, stable, phalloidin-stainable actin filaments are also not present in normal mammalian nuclei, however exceptions include baculovirus infection, antisynthase syndrome, and intranuclear rod myopathy (Charlton and Volkman, 1991; Goley et al., 2006; Domazetovska et al., 2007a; Stenzel et al., 2015). Furthermore, expression of certain actin bundling proteins such as α-catenin (Daugherty et al., 2014; McCrea and Gottardi, 2016), supervillin (Wulfkuhle et al., 1999), myopodin (Weins et al., 2001), c-Abl (Aoyama et al., 2013), mutant espins (Loomis et al., 2006), and expressing nuclear targeted actin all form stable, phalloidin-stainable actin filaments (Kokai et al., 2014). Similarly, knockdown of the actin depolymerization factor MICAL-2 (Lundquist et al., 2014) or the nuclear actin export factor, exportin-6 (Dopie et al., 2012) induce formation of phalloidin-stainable nuclear actin filaments. While formin-dependent nuclear actin filaments that form in response to serum stimulation and cell spreading are reported to be highly transient (Baarlink et al., 2013; Plessner et al., 2015), the phalloidin-stainable nuclear actin filaments that form under the above conditions are highly stable and may be detrimental to the cell as is the case in patients with the skeletal muscle disease, intranuclear rod myopathy (Vandebrouck et al., 2010; Serebryannyy et al., 2016b).

Over 180 different mutations occur in the skeletal α-actin gene (Laing et al., 2009; Ravenscroft et al., 2011); most are dominant and *de novo*. Skeletal myopathies are usually severe, affect many actin functions, and can lead to premature death. Previous experiments have established that certain mutant actins translocate to the nucleus and form stable, phalloidin-stainable actin filaments in the nucleus (Costa et al., 2004; Ilkovski et al., 2004). The formation of nuclear actin filaments in skeletal muscle causes the human disease intranuclear rod myopathy (Jenis et al., 1969; Goebel and Warlo, 1997). Patients with intranuclear rod myopathy usually exhibit a severe clinical phenotype with fatalities caused by diaphragmatic weakness and respiratory failure (Goebel and Warlo, 1997; North et al., 1997; Laing et al., 2009). These intranuclear actin aggregates stain with phalloidin and α-actinin, but rarely with cofilin (Vandebrouck et al., 2010). The actin mutatants that cause intranuclear rod myopathy also demonstrate decreased incorporation into muscle thin filaments (Costa et al., 2004; Ilkovski et al., 2004; Domazetovska et al., 2007a,b). Despite maintaining contractile function, patients continue to experience severe muscle weakness, and cell culture models exhibit a decrease in mitotic index (Domazetovska et al., 2007a,b). Further, mutations in α-actin that cause intranuclear rod myopathy have been suggested to alter serum response factor signaling (Visegrády and Machesky, 2010). These studies implicate an alternate role for actin and suggest a pathogenesis aside from decreased contractility.

Nuclear actin structures have been observed repeatedly in a variety of circumstances. Yet, the function of nuclear actin polymerization and the different nuclear actin structures described above is unknown. Therefore, we sought to determine what effects the formation of nuclear actin structures has on the nucleus. Using cofilin/actin rods and models of intranuclear rod myopathy, we find nuclear actin structures are able to change the localization of RNAPII and chromatin, potentially contributing to their pathogenesis.

## Results

### Organization of RNAPII and chromatin in cells with cofilin/actin rods

**Figure 1:**
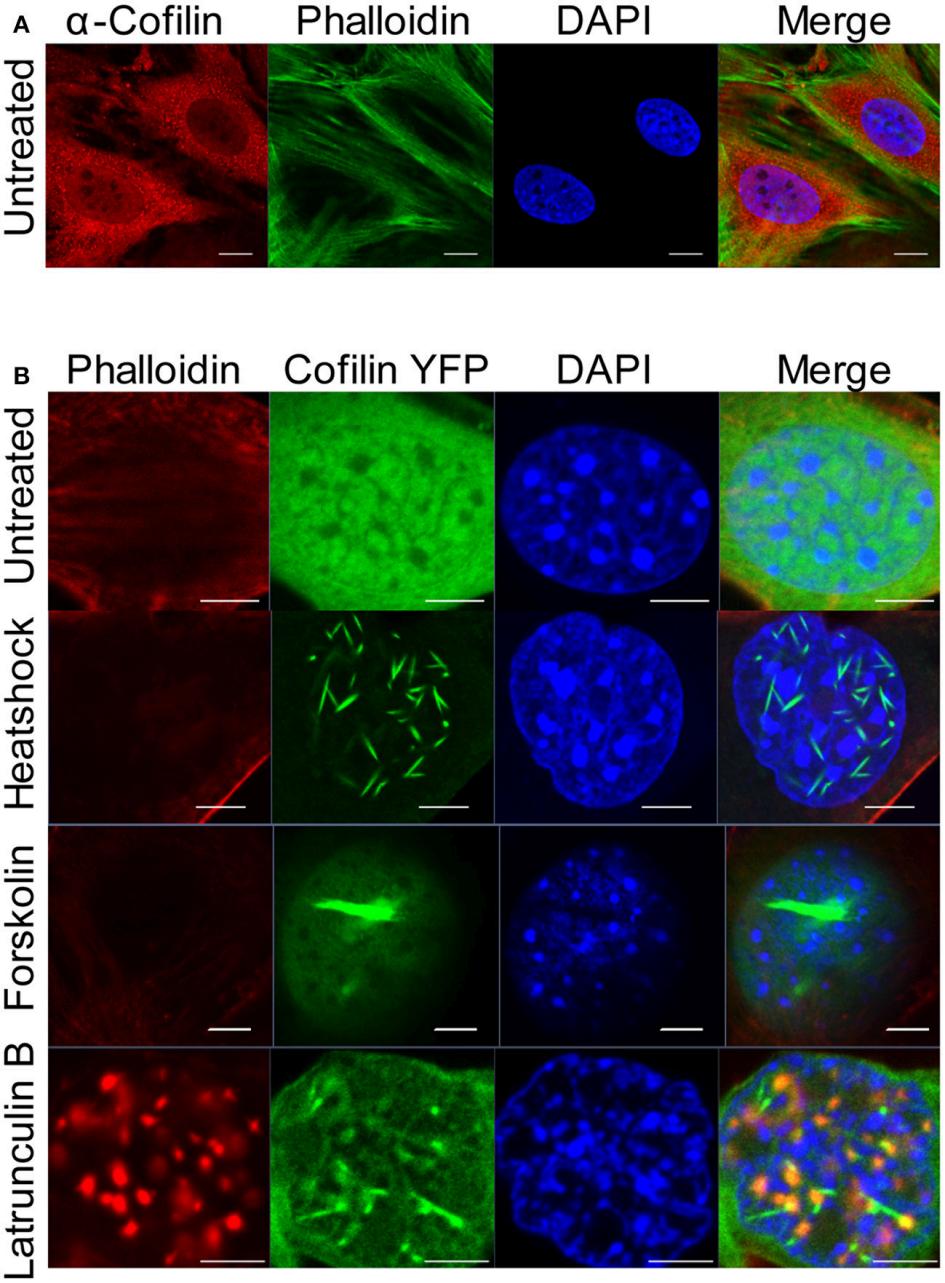
**Cofilin/actin rod formation in the presence of cellular stress. (A)** STHdh cells were stained for endogenous actin filaments using phalloidin (green), cofilin (red) and DAPI (blue). Scale bars = 10 μm. **(B)** STHdh cells stably expressing cofilin YFP (green) were left untreated, heatshocked at 42°C for 1 h, treated with 10 μM forskolin overnight, or treated with 1 μg/mL latrunculin B for 1 h. Cells were stained with phalloidin (red) and DAPI (blue). Scale bars = 2 μm.

**Figure 2:**
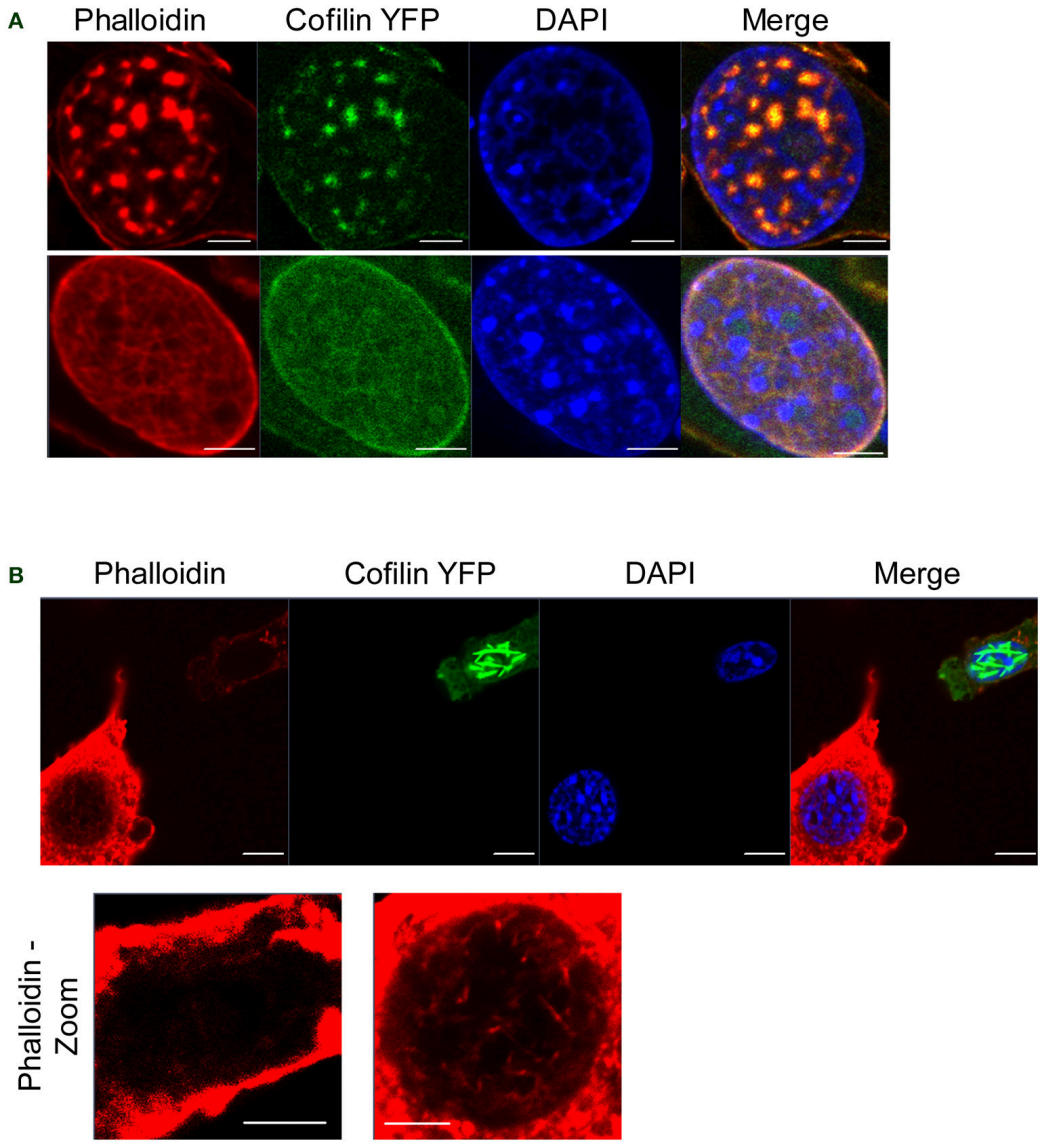
**Cofilin/actin rod formation and phalloidin-stainable nuclear actin filament formation in response to heatshock. (A)** STHdh cells expressing cofilin YFP (green) were heatshocked at 42°C for 1 h in PBS without serum, fixed, and stained for phalloidin (red). Note the presence of actin structures that label with both phalloidin and cofilin (top and bottom). Scale bars = 2 μm. **(B)** Same as in **(A)** except each neighboring cell exhibits either cofilin/actin rods (top right) or phalloidin-stainable filaments (bottom left), suggesting these different structures are able to form under the same conditions. Scale bars = 10 μm; zoom = 5 μm.

To investigate the consequences of nuclear actin polymerization on chromatin and RNAPII, we first examined how different acute cellular stresses, which have been documented to form nuclear cofilin/actin rods, affect a mouse neuronal striatal cell line (STHdh) stably expressing cofilin YFP (Munsie et al., 2011). Under normal culture conditions, STHdh cells do not exhibit phalloidin- nor cofilin-labeled actin filaments within the nucleus (Figure 1A). However, with certain stimuli such as heatshock, forskolin treatment, or treatment with the actin depolymerization drug, latrunculin B, a population of STHdh cells formed cofilin-labeled rods which did not stain with phalloidin (Figure 1B). Latrunculin B treatment resulted in some co-localization between cofilin/rods and phalloidin labeling in large puncta within the nucleus. Additionally, we found heatshock performed in Dulbecco's phosphate-buffered saline (PBS) lacking fetal bovine serum and supplemented with CaCl_2_ (0.9 mM) and MgCl_2_ (0.5 mM) resulted in a population of cells with nuclear filaments that stained with phalloidin and incorporated cofilin (Figure 2A) as well as neighboring populations that exhibited only cofilin/actin rods or phalloidin-stainable filaments but not both (Figure 2B). This suggests that the extracellular environment and intrinsic properties of each cell may modulate the type of nuclear actin structure formed.

**Figure 3:**
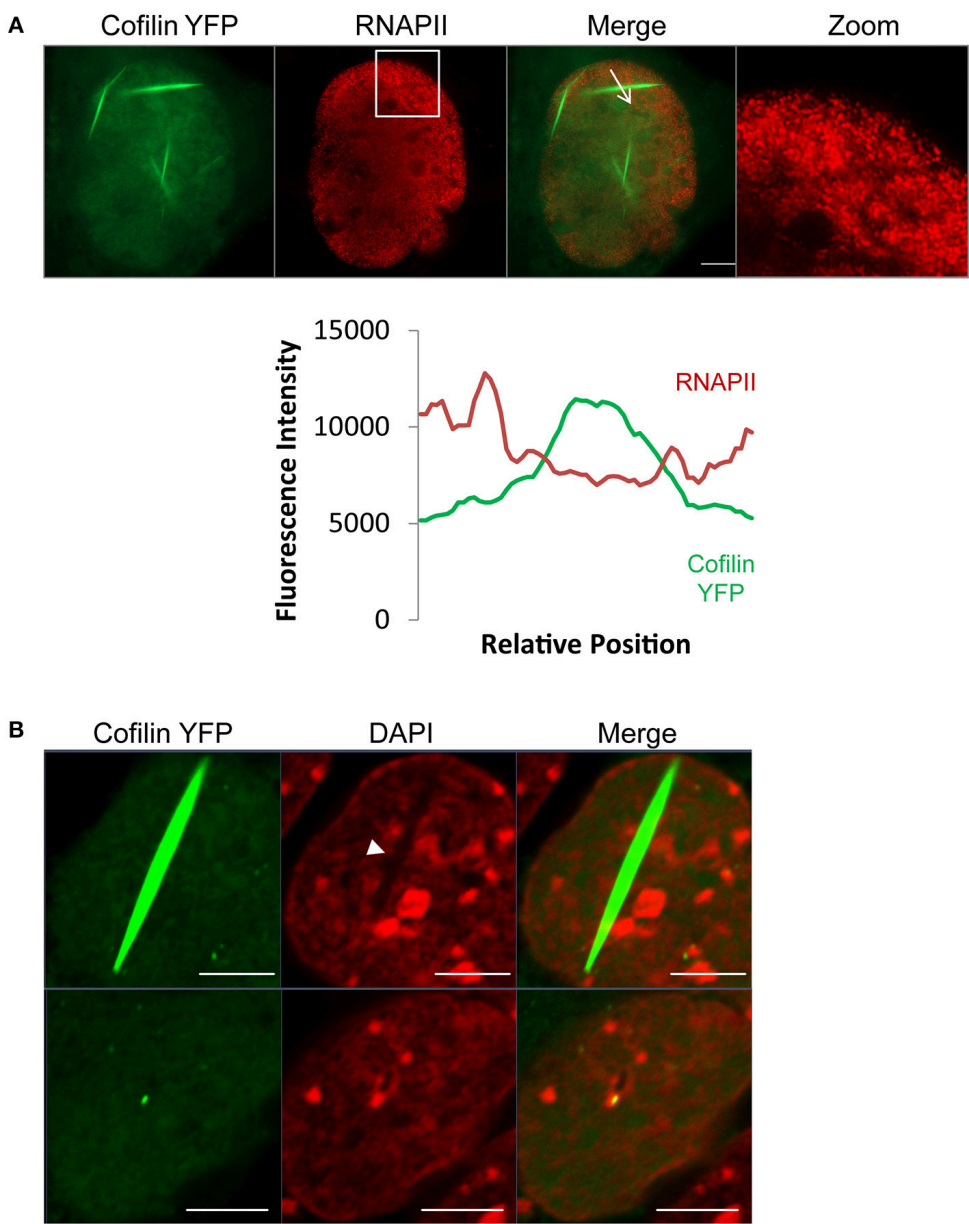
**Cofilin/actin rods displace RNAPII and chromatin. (A)** STHdh cells expressing cofilin YFP (green) were heatshocked at 42°C for 1 h, fixed, and stained for RNAPII (red). Formation of cofilin/actin rods due to acute cellular stress reorganizes RNAPII localization. Fluorescence intensity plot shows the inverse correlation between RNAPII and cofilin localization (plotted fluorescence trace is indicated in the image by the arrow). Box indicates zoomed area. **(B)** STHdh cells stably expressing cofilin YFP (green) were treated overnight with 10 μM forskolin, fixed, and labeled with DAPI (red). Shown are two nuclei from the same field of view. Note the redistribution of chromatin in the nucleus with the cofilin/actin rod (arrowhead). Scale bars = 5 μm.

To determine if cofilin/actin rods have an effect on the organization of the nucleus, we induced rod formation in STHdh cells, fixed, then stained these cells with RNAPII antibodies (Figure 3A). Superresolution structured illumination microscopy (SIM) showed that RNAPII is redistributed away from areas with cofilin/actin rods. Furthermore, confocal microscopy of cofilin/actin rods in forskolin-treated STHdh cells similarly showed cofilin/actin rods had redistributed DAPI staining, suggesting a change in chromatin distribution (Figure 3B). Therefore, we conclude nuclear cofilin/actin rods cause a change in nuclear topography.

### Nuclear organization in a cell model of intranuclear rod myopathy

**Figure 4:**
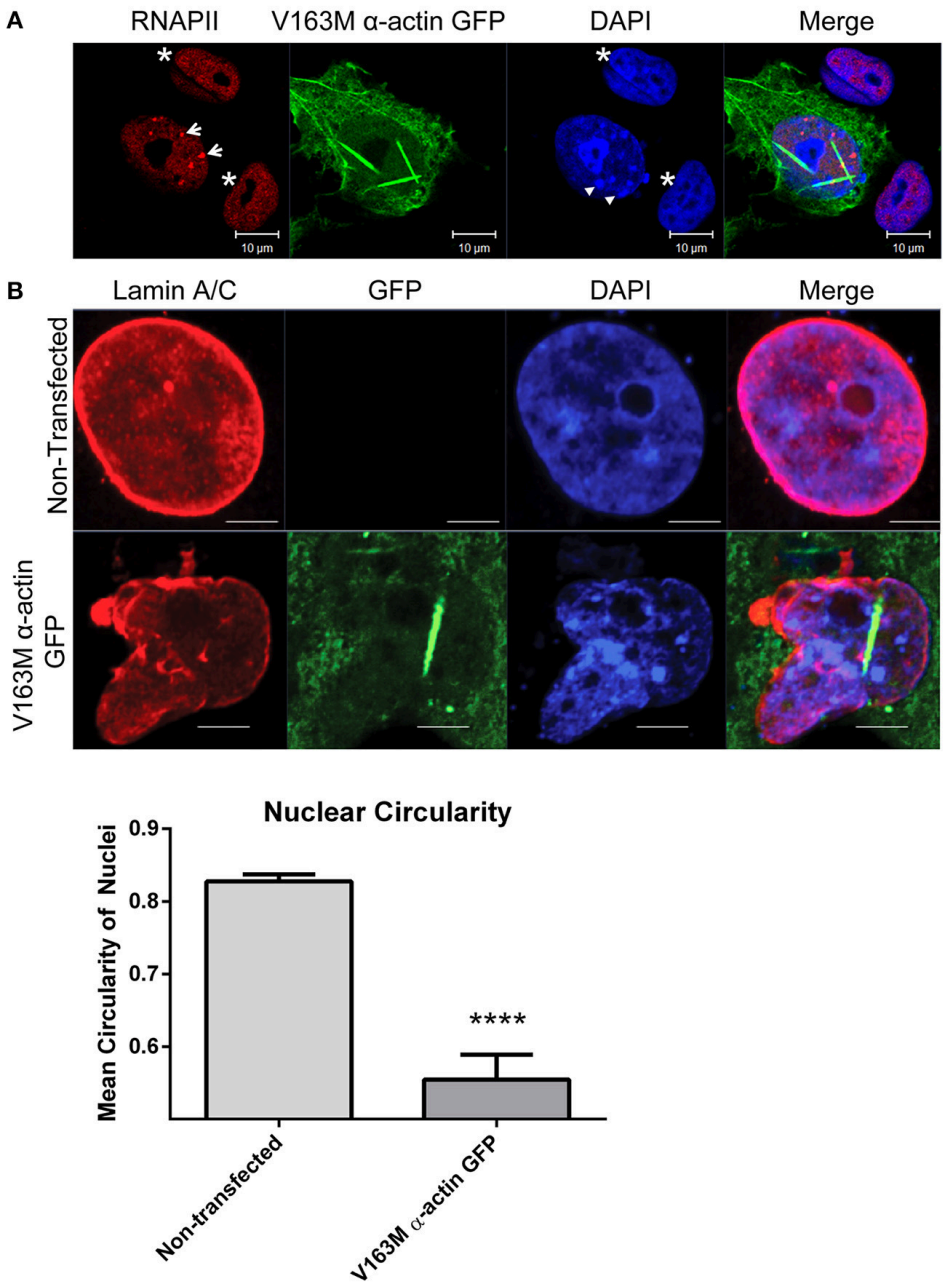
**Cell culture model of intranuclear rod myopathy exhibits aggregation of RNAPII, chromatin and changes in nuclear structure. (A)** COS7 cells transfected with V163M α-actin GFP (green) for 48 h were fixed and stained with RNAPII (red) and DAPI DNA stain (blue). Non-transfected cells are shown as controls (asterisks). Note the altered distribution of both RNAPII (arrows) and chromatin (arrowheads) in cells with nuclear actin filaments. **(B)** COS7 cells were untransfected or transfected with V163M α-actin GFP (green) for 48, fixed, and stained for lamin A/C (red). Nuclear circularity was quantified as a ratio of the length of the Y-axis:X-axis in Image J, where a value of 1 is considered a perfect circle. *N* = 10 for Non-transfected; *N* = 3 for V163M α-actin GFP. At least 60 cell were counted in each group. ^****^ *P* < 0.0001 by *t*-test.

We have recently shown the formation of stable, phalloidin-stainable nuclear actin filaments alters RNAPII localization using several cell culture models (Serebryannyy et al., 2016b). This included the V163M mutation in skeletal α-actin that causes intranuclear rod myopathy (Ilkovski et al., 2004; Domazetovska et al., 2007a,b). We found that nuclear actin polymerization reduced the monomeric actin pool and correlated with decreased proliferation and transcription in culture and *in vitro* (Serebryannyy et al., 2016b). Consistent with previous work (Domazetovska et al., 2007a; Serebryannyy et al., 2016b), we find expression of V163M α-actin GFP in COS7 cells causes formation of actin filaments in the nucleus that grow with time but are restricted by the nuclear periphery (Video S1). Using confocal microscopy, we find that the presence of nuclear actin filaments induced by V163M α-actin GFP correlates with changes in RNAPII localization into clusters as well as changes in chromatin organization as marked by DAPI staining (Figure 4A). This is consistent with the recent finding that monomeric nuclear actin can inhibit nuclear histone deacetylase activity (Serebryannyy et al., 2016a). Nuclear actin polymerization reverses this inhibition and correlates with more condensed chromatin. Furthermore, immunostaining for lamin A/C showed that cells with nuclear actin filaments exhibited defects in nuclear structure and had misshapen nuclei (Figure 4B).

### Organization of RNAPII and chromatin in human intranuclear rod myopathy tissue

**Figure 5:**
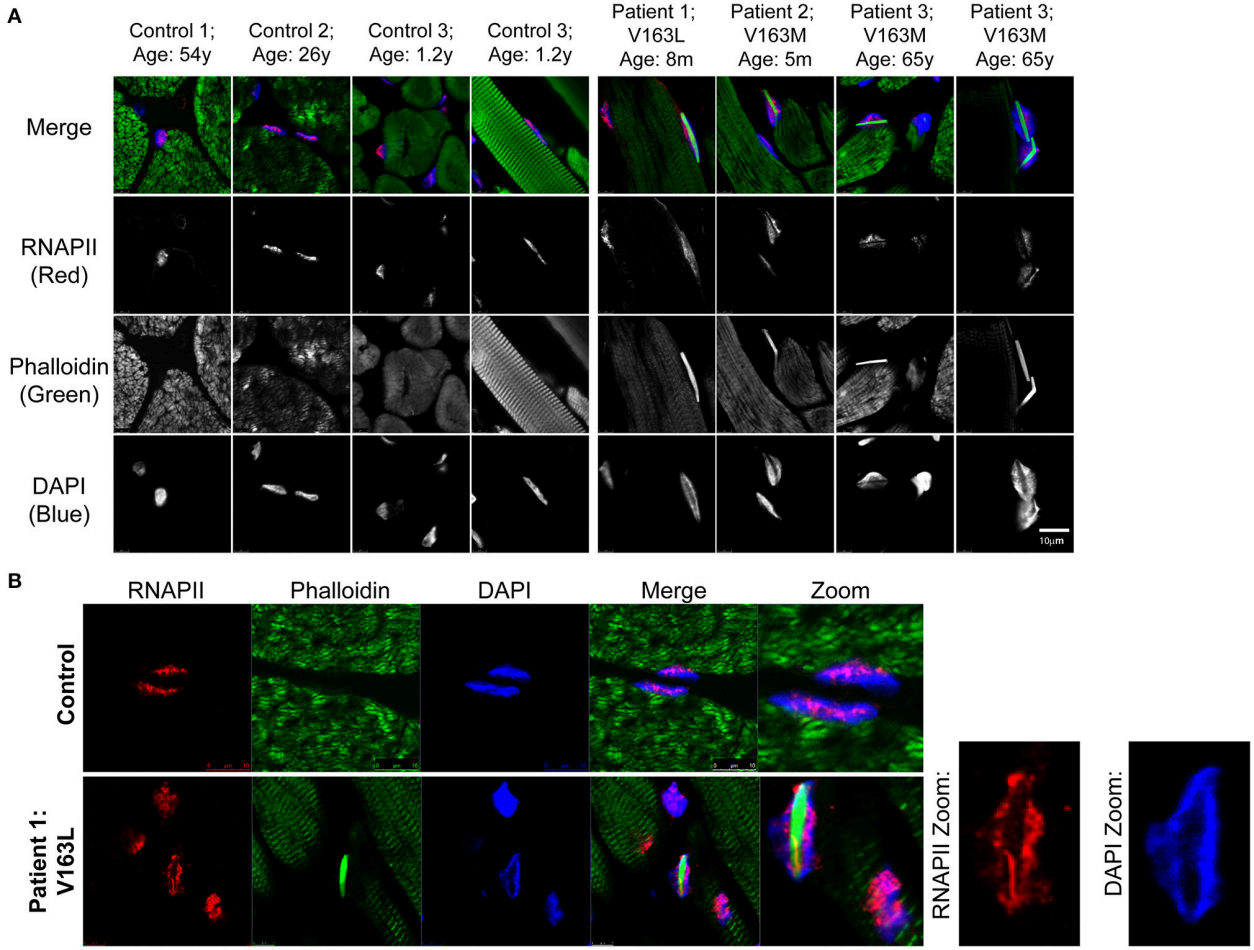
**Patient intranuclear rod myopathy tissue shows nuclear actin filaments displace RNAPII and chromatin. (A)** Age-matched healthy subjects' skeletal muscle tissue or skeletal muscle tissue from human patients diagnosed with intranuclear rod myopathy (V163M and V163L α-actin mutations) was stained with phalloidin to recognize actin filaments (green), RNAPII (red) and DAPI (blue). Additional images of sections from intranuclear rod myopathy patients and control subjects stained with phalloidin can be found in (Domazetovska et al., 2007b). **(B)** Staining same as in **(A)** of sections from Patient 1 (V163L α-actin); zoomed panels show how the large actin filaments occupy much of the nuclear space and result in the displacement of both RNAPII and chromatin to the nuclear periphery.

The geometrical constraints and 3D environment of cells grown on culture dishes are different than cells within tissues (Pampaloni et al., 2007). Therefore, we sought to examine the nuclear topography of human tissues with nuclear actin filaments. Frozen tissues from patients diagnosed with intranuclear rod myopathy due to V163M or V163L mutations in α-actin and age-matched control subjects were examined for changes in RNAPII and chromatin. Tissue was stained with phalloidin to identify intranuclear rods, RNAPII, and DAPI to label chromatin (Figure 5A). Strikingly, we see nuclear actin filaments in intranuclear rod myopathy patient tissues are more prominent than those seen in COS7 cells. Furthermore, the formation of these filaments displaced both RNAPII and chromatin (Figures 5A,B), likely influencing transcriptional regulation in these cells. Because nuclear compaction in these tissues was already high, it was difficult to document more subtle effects such as changes in nuclear shape or RNAPII aggregation as seen in our cell culture model (Figure 4). Nevertheless, it was clear that nuclei with actin filaments had obvious defects in their topology, suggesting this effect may contribute to the pathology of intranuclear rod myopathy.

## Discussion

Previous studies have shown nuclear actin regulates multiple transcription signaling pathways, chromatin remodelers, and general transcription by all three RNAPs (de Lanerolle and Serebryannyy, 2011; Grosse and Vartiainen, 2013; Kapoor and Shen, 2014). This raises the question of the impact of nuclear actin rods or filaments on transcription. While cellular stressors, such as heatshock, can induce the formation of cofilin/actin rods, α-actin mutations implicated in intranuclear rod myopathy induce the formation of phalloidin-stainable filaments in the nucleus. Not only is the composition of these structures different (i.e., the presence of cofilin vs. phalloidin staining), but they can also alter the nucleus to different extents (i.e., RNAPII/chromatin displacement vs. aggregation), and the properties of these structures vary with cell and tissue type. Despite this heterogeneity, we find that the formation of actin structures within the nucleus leads to alterations in the organization of the nucleus.

It is easy to envision how obtrusive nuclear actin structures could impede proper genetic regulation; however, it is unclear if these structures serve a functional purpose or are a consequence of the disease. Notably, stressors such as heatshock have been shown to cause changes in the cell's transcription program (Morimoto, 1998) and alter RNAPII clustering (Cisse et al., 2013). Our study suggests nuclear actin may play a role in these responses. It has been hypothesized that the purpose of cofilin/actin rods are protective and may form to regulate ATP levels by limiting actin treadmilling (Whiteman et al., 2009; Munsie and Truant, 2012). If these rods also impair transcription, it may be another method of resource conservation during stress. Interestingly, wildtype α-actin also forms nuclear actin filaments in response to hypoxia and treatment with actin depolymerizing drugs (Domazetovska et al., 2007a). Careful cell manipulations and new age techniques such as laser dissection of the nucleus (Paz et al., 2013) may help answer what role these nuclear actin structures play, if certain nuclear proteins or regions of chromatin have preferential association with these structures, and ultimately, how these nuclear actin structures may affect gene expression.

The different stimuli and cell types used in this study also highlight the phenotypic heterogeneity of nuclear actin polymerization. Why different media conditions can induce the formation of cofilin/actin rods or phalloidin-stainable filaments is curious (Figures 1, 2). Additionally, why neighboring cells exhibit different nuclear actin structures when confronted with the same stress (Figure 2) remains an outstanding question. Heterogenic responses exist not only between the types of structures that occur, but also between cell types. It is unclear why neurons seem to be the most sensitive cell type to form cofilin/actin rods both in the nucleus and in the cytoplasm, as seen in Alzheimer's and other neuronal pathologies (Minamide et al., 2000; Bamburg and Wiggan, 2002; Huang et al., 2008; Whiteman et al., 2009; Bamburg et al., 2010; Munsie et al., 2011). These differences could depend on cytoskeleton composition, the balance of actin polymerizing and depolymerizing factors in the nucleus, differences in import and export rates, or perhaps be a consequence of the post-mitotic nature of neurons and myocytes. Notably, modulators of cofilin phosphorylation have been implicated in mediating nuclear actin translocation (Dopie et al., 2015) and may therefore be a point of regulation. Future studies to delineate the roles of actin binding proteins in the nucleus may offer new insights into why different actin structures form in the nucleus in response to different stimuli, what regulates the size and composition of these structures, and what functions nuclear actin structures may serve.

Similarly, it is perplexing that certain cell types such as cardiomyocytes resist nuclear actin polymerization. Intranuclear rods do not appear to form in cardiac tissue (North et al., 1997), despite expressing skeletal α-actin during development (Ruzicka and Schwartz, 1988). Further, of the relatively few mutations documented to occur in cardiac α-actin, none have been shown to cause nuclear actin polymerization. Understanding the pathological consequences of these different nuclear actin structures (e.g., chromatin landscape changes or nuclear actin polymerization) in different tissues may have implications in understanding the role of actin in the nucleus, how cells respond to stress, and why pathological nuclear actin filaments are detrimental.

## Materials and methods

Skeletal muscle tissue from one patient with an ACTA1 V163L mutation, two patients with ACTA1 V163M mutations, and age-matched controls with unrelated disorders were obtained during routine diagnostic procedures (Ilkovski et al., 2004; Domazetovska et al., 2007b). This research was approved by the Human Research Ethics Committee of the Children's Hospital at Westmead, Australia (10/CHW/45) and performed with patient consent.

### Cell culture and treatments

COS7 were cultured in Dulbecco's modified minimum essential medium (DMEM; Corning) with 4.5 g/L glucose and L-glutamine, without sodium pyruvate, supplemented with 10% fetal bovine serum (FBS; Sigma-Aldrich), penicillin and streptomycin (Gibco) in a humidified atmosphere containing 5% CO_2_ at 37°C. STHdh and STHdh cells stably expressing cofilin YFP (Ray Truant, McMaster University) were cultured in DMEM with 10% FBS, 1% penicillin, and streptomycin as well as 400 μg/mL G418 (Enzo Life Sciences) at 33°C. To heatshock, STHdh cells in DMEM or Dulbecco's Phosphate-Buffered Saline with calcium and magnesium (PBS; Corning) were paraffin wrapped and submerged into a waterbath held at 42°C for 1 h then immediately fixed in 4% paraformaldehyde. For forskolin and latrunculin B (Calbiochem) treatment, cells were plated in 12-well plates and treated with 10 μM forskolin overnight or 1 μg/mL latrunculin B for 1 h at 33°C in DMEM. Cell transfections were carried out using Polyjet transfection reagent (SignaGen) according to the manufacturer's protocol. V163M α-actin GFP was a gift from Dr. Kathryn North (University of Sydney).

### Immunostaining and microscopy

Frozen skeletal muscle tissue was cut in 8 μm slices and fixed with 3% PFA in PBS for 10 min followed by permeabilization in 0.5% triton X for 8 min and three washes in PBS. Slides where then incubated in blocking solution (2% BSA) for 15 min. Primary antibody (1:50 dilution of RNA polymerase II antibody, 4H8, ab5408, Abcam) was incubated in blocking solution for 2 h at room temperature. After primary antibody incubation, the tissue sections were washed three times in PBS for 10 min each. Secondary Alexa555-conjugated anti-mouse antibody (Life Techology) diluted 1:200 in blocking solution, Alexa488-conjugated Phalloidin (1:40, Life Technology) and DAPI were applied for 2 h at room temperature. Finally, sections were washed as above, mounted in Vectashield and imaged using an SP5 confocal microscope. Results were consistent among the three intranuclear rod myopathy patients shown (at least 4 images were obtained for each patient).

For immunocytochemistry, cells were plated on glass coverslips at least 24 h before fixation or transfection. V163M α-actin GFP transfection efficiency in COS7 cells at 48 h was 50.4 ± 15.2%. Of the transfected cells, 52.1 ± 6.7% exhibited nuclear actin filaments, and of the population with nuclear actin filaments 65.3 ± 1.9% exhibited changes in RNAPII and chromatin distribution as scored in two experiments (total of 144 and 72 cells; mean ± standard deviation). Cells were fixed in 4% PFA for 10 m then permeabilized with 0.3% Triton X100 (Sigma-Aldrich) in PBS for 7 m. After permeabilization, cells were washed with PBS and incubated in 2% BSA in PBS for 1 h at room temperature. Cells were stained using a humidity chamber. Primary antibody was added for 1 h at room temperature or overnight at 4°C. Cells were then washed with PBS and secondary antibody was added for 1 h at room temperature. Cells were washed a final time and mounted using Vectashield containing DAPI. Primary antibodies include RNA polymerase II (4H8, Abcam; 1:200), actin (C4, EMD Millipore; 1:100), cofilin (Sigma-Aldrich; 1:10,000), lamin A/C (Santa-Cruz; 1:100) and phalloidin (Invitrogen; 1:400).

Immunocytochemistry samples were examined using a Zeiss 710 Laser Scanning confocal microscope and single confocal slices were taken through the nucleus or using a Nikon N-SIM microscope (Northwestern University Nikon Imaging Center). The SIM fluorescence intensity plot was generated using Nikon NIS-Elements software where fluorescence signal intensity was plotted along the selected region (relative distance). Live cell imaging was performed using an Olympus VivaView fluorescence microscope. Image files were processed with Zen software or Image J.

To quantify nuclear circularity, cells were left untransfected or transfected for 48 h with V163M α-actin GFP, fixed, and stained with lamin A/C antibody as described above. Confocal images were acquired through the nucleus. To quantify nuclear circularity, Image J was used to threshold the images and circularity was calculated as the ratio of the lengths of Y axis:X axis with a value of 1 representing a perfect circle.

## Author contributions

LAS, MY, STC, and PdL: Designed the experiments; LAS, MY, and MP: Performed the experiments. LAS and PdL: Wrote the manuscript.

### Conflict of interest statement

The authors declare that the research was conducted in the absence of any commercial or financial relationships that could be construed as a potential conflict of interest.

## Supplementary material

Video S1 **Formation of V163M α-actin GFP filaments**. COS7 cells transfected with V163M α-actin GFP were imaged over 22 h at 30 minute intervals.
